# Infection with Trematodes in *Littorina obtusata* Snails (Gastropoda: Littorinidae) with Different Shell Color Genotypes

**DOI:** 10.1134/S0012496623700448

**Published:** 2023-10-13

**Authors:** E. V. Kozminsky

**Affiliations:** Zoological Institute, Russian Academy of Sciences, St. Petersburg, Russia

**Keywords:** shell color, *Littorina obtusata*, infection with trematodes, genotype, phenotype

## Abstract

The prevalence of infection with trematode parthenitae was studied in *Littorina obtusata* littoral periwinkles with different shell color genotypes. Activities of genes responsible for a purple or orange single-pigment background shell coloration was not found to affect the prevalence of trematode infection in periwinkles. In *L. obtusata* with a yellow-purple background shell color and a pattern of white pigment spots on the shell, the prevalence of infection with *Microphallus piriformes* and *M. pygmaeus* was lower than the theoretical expectation. The prevalence of infection in periwinkles with purple, orange, or white stripes on the shell did not differ from that of unstripped periwinkles. The differences found were presumably associated with genetically determined susceptibility to infection. Possible consequences of differential infection with trematodes in periwinkles with different shell color genotypes are discussed in terms of the stability of parasitic systems and possible changes in the phenotypic structure of the host population.

## INTRODUCTION

Shell color polymorphism is a common phenomenon in mollusks [1]. Given that pleiotropic effects are exerted by genes, phenotypic traits often mark the physiological differences in responses to various environmental factors between individuals [2, 3]. Parasites are among the most important and widespread factors that affect animal populations. A variation in susceptibility to infection between morphs leads to differential infection of individuals in a host population and acts as a factor that contributes to the stability of parasitic systems. On the other hand, complete parasitic castration of the host is often caused by infection and may directly affect the phenotypic structure of the host population.

The periwinkle *Littorina obtusata* (Linnaeus, 1758) from the White Sea provides a convenient model to study the parasite–host interactions at the population level and is characterized by substantial polymorphism of shell color [4–6]. Phenotypically different periwinkles have been observed to have physiological differences in their responses to salinity, temperature, drying time, and certain other factors [7–9]. *Littorina obtusata* serves as the first intermediate host of several trematode species [10–17] and, in particular, microphallids of the pygmaeus group (Trematoda: Microphallidae), which cause severe diseases in waterfowl [18, 19]. Infection with trematodes causes complete parasitic castration in snails [20–22], and this circumstance may determine a directional change in snail population structure.

Sergievsky [23] has studied the variation in the prevalence of infection in *L. obtusata* periwinkles with different phenotypes from norther Russian seas. Ample new data on the formation and inheritance of shell color traits in *L. obtusata* have been reported in the past years [6, 24–27], making it possible to evaluate the association with infection prevalence not only for the phenotype, but also for the genotype.

The objective of this work was to study the specifics of infection with trematode parthenitae in *L. obtusata* (Linnaeus, 1758) periwinkles differing in shell color genotype.

## MATERIALS AND METHODS

*Littorina obtusata* (Linnaeus, 1758) is a widespread littoral snail and is widely found from zero depths to the upper limit of areas inhabited by the brown alga *Fucus vesiculosus* Linnaeus, 1753. The species is diclinous. The shell height is no more than 12 mm and the maximal diameter, 10 mm in periwinkles of the White Sea. The average lifespan is five years; the maximal lifespan is approximately 10 years [28].

Shell color traits are highly polymorphic in *L. obtusata* [4–6]. Four pigments are involved in producing the shell color: melanin, two carotenoids, and (presumably) guanine [6]. Melanin is responsible for a brown (conventionally termed purple) color of shell regions; carotenoids determine yellow and orange colors; and guanine determine a white color. Various coloration elements can develop on the basis of the same pigment.

The background shell color forms as one, two, or three pigments are included in the shell [6].^1^ Depending on the pigments included in the shell, different background coloration variants are observed: monochromatic (yellow, orange, or purple), dichromatic (two-layer yellow-purple, yellow-orange, orange-purple, and white-purple), and trichromatic (three-layer yellow-orange-purple, yellow-white-purple, or orange-white-purple). In terms of genetics, inclusion of each pitment in the shell is a conventionally simple trait [29] and is controlled by a separate group of genes, which are related to synthesis, transport, and shell distribution of the pigment. When the background shell color forms, at least two complementary genes determine the inclusion of the purple and yellow pigments in the shell, and at least one gene determines the inclusion of the orange pigment [26]. The inclusion of each pigment in the shell is a dominant trait. Only a single gene group is active when the monochromatic variants form; two or three gene groups work to form the two-pigment or three-pigment variants, respectively.

The white pigment is responsible for the formation of a spotted pattern on the shell. Particular elements of the pattern are lens-shaped pigment inclusion regions which form in the upper part of the irregular prismatic shell layer [6]. A mutual arrangement of pattern elements and the degree of their fusion vary greatly. At least two complementary genes are responsible for the presence of a pattern [24]. A present pattern is a dominant trait. Broad longitudinal stripes form as bands of the purple, white, and orange pigments [6, 25, 27]. A single biallelic gene determines the formation of a particular stripe type. The presence of purple or white stripes is a dominant trait, while the presence of orange bands is most likely a recessive trait.

The majority of coloration traits are inherited independently, while linked loci are responsible for the formation of purple stripes and the inclusion of the orange pigment in the shell (Kozminsky, unpublished data).

Data for the study were collected while monitoring the *L. obtusata* population dynamics on the western bar of the Yuzhnaya Bay of the Ryazhkov Island (Kandalaksha State Nature Reserve, 67^o^00′N, 32^o^34′E) from 2001 to 2016.

Material was collected once yearly, in late August or early September. Quantitative collections were obtained from areas of 1/40 m^2^, which were arranged in series of three areas each along a transect at depths of 0, 5, 10, 15, 20, and 25 m. The samples were transported to the lab, washed with fresh water in a 0.6-mm sieve, and examined. In each *L. obtusata* periwinkle, the maximal diameter of the shell was measured, shell coloration traits were recorded, and examination for infection with trematode parthenitae was performed via dissection.^2^ Periwinkles with a shell diameter of at least 3 mm were selected for further analysis because the phenotype and infection could not be established reliably at a lower shell diameter. The periwinkle sample sizes are summarized in Table 1.

**Table 1.** Probability values corresponding to the χ^2^ values calculated in comparisons of the observed and expected prevalences of infection for *L. obtusata* periwinkles with different genotypes

Year	Background color involving yellow pigment					White-spotted pattern					*N*
	MCR	MPIR	MPSD	MPYG	MTRI	MCR	MPIR	MPSD	MPYG	MTRI	
2001	**0.037**	**0.009**	0.595	0.526	–	0.868	0.825	**0.034**	0.371	–	338
2002	**0.046**	**0.020**	0.216	0.320	0.756	0.172	0.722	0.440	0.265	**0.010**	878
2003	**0.001**	**0.037**	0.496	**0.025**	0.592	0.140	0.167	0.625	0.892	0.072	583
2004	**0.039**	**0.010**	0.246	0.403	0.766	0.233	0.258	0.275	0.268	0.764	722
2005	**0.002**	0.180	**0.013**	**0.001**	0.880	**0.018**	**0.032**	0.132	0.568	0.768	396
2006	0.451	0.103	0.255	**0.004**	–	0.187	0.464	0.089	**0.007**	–	368
2007	**0.014**	0.092	0.148	–	–	0.125	0.492	0.148	–	–	314
2008	0.399	0.622	0.194	0.125	–	0.796	0.413	0.202	0.118	–	337
2009	0.319	0.587	0.444	**0.003**	0.641	0.158	0.255	0.648	0.702	0.748	479
2010	0.131	0.804	0.742	**0.003**	–	**0.038**	0.309	0.953	**0.024**	–	558
2011	**0.015**	**0.024**	0.521	**0.036**	0.661	0.080	0.306	0.384	0.071	0.469	640
2012	0.477	**0.024**	0.076	0.784	0.706	0.061	**0.027**	0.508	0.133	0.721	515
2013	0.537	0.657	0.358	0.239	–	0.254	0.768	0.474	0.153	–	562
2014	0.141	**0.018**	0.765	0.765	–	0.279	0.464	0974	0.351	–	254
2015	**0.006**	0.237	**0.049**	**0.045**	**0.031**	0.273	0.193	0.960	0.193	0.513	482
2016	**0.027**	0.257	0.767	0.257	–	0.290	0.628	0.808	0.628	0.808	384

Values significant at a 5% significance level are in bold. MCR, all microphallids (including parthenitae of unidentified species of early developmental stages); MPIR, *Microphallus piriformes*; MPSD, *M. pseudopygmaeus*; MPYG, *M. pygmaeus*; MTRI, *M. triangulatus*. *N* is the sample size.

In the habitat examined, *L. obtusata* acts as the first intermediate host for nine trematode species: *Microphallus piriformes*, *M. pygmaeus*, *M. pseudopygmaeus*, *M. triangulatus*, *Podocotyle atomon*, *Cryptocotyle lingua*, *Tristriata anatis*, *Himasthla elongate,* and *Renicola* sp. The four last species are found occasionally; their invasion rates do not exceed 0.5%. Although *P. atomon* is a common species, considerable differences in the prevalence of infection were not detected between periwinkles with different genotypes in a preliminary analysis. Differences in infection prevalence were therefore studied only in *L. obtusata* infected with microphallids of the pygmaeus group.

Periwinkles were sorted into groups differing in activities of genes responsible for different elements of shell coloration in the analysis. Three first groups included the periwinkles that displayed activities of the genes responsible for background colors involving the purple (purple, yellow-purple, white-purple, orange-purple, etc. colors), yellow (yellow, yellow-purple, yellow-orange, etc. colors), and orange (orange, yellow-orange, orange-purple, etc. colors) pigments. A fourth group included the periwinkles that had active genes responsible for the formation of a white-spotted pattern on the shell. Three other groups included the periwinkles that had active genes responsible for the formation of purple, white, and orange broad longitudinal stripes on the shell.

The results were statistically analyzed using the conventional statistical methods. In the case of genotypes associated with the background coloration and a spotted pattern, a fourfold table of observed and expected numbers was constructed for each separate year.

In the case of genotypes responsible for the formation of broad longitudinal stripes, the respective samples were small and were therefore pooled over all study years for each genotype. Expected numbers for corresponding cells of the table were calculated by multiplying the respective relative marginal frequencies by the sample size. Then the χ^2^ test was used to compare the expected and actual prevalence values in periwinkles with active or inactive genes responsible for a particular shell coloration trait. When a sample was small, χ^2^ values were corrected for small theoretically expected numbers [31]. An observed-to-expected prevalence ratio was calculated to present the results in an illustrative form; the ratio was equivalent (mathematically) to a ratio of invasion intensity in the given group to the average invasion intensity in the population. The prevalence of infection tended to be lower than expected in certain cases over several years. Although insignificant statistically, systematic shifts of the kind testify again that differences exist. The probability for a series of *n* tests to include *m* or more observations where the observed numbers are lower than expected and *n – m* observations where the observed numbers are higher than or equal to the expectation was calculated from the following equation:

$$P = \sum\limits_m^n {\left[ {\left( {\begin{array}{*{20}{c}} m \\ n \end{array}} \right){{p}^{m}}{{q}^{{\left( {n - m} \right)}}}} \right]} {{\;}} = \sum\limits_m^n {\left[ {\left( {\begin{array}{*{20}{c}} m \\ n \end{array}} \right){{{\left( {1{\text{/}}2} \right)}}^{n}}} \right]} {{\;}}{\text{,}}$$

where *p* is the probability for the observed numbers to be lower than expected and *q* is the probability for the observed numbers to be higher than or equal to the expectation (it is assumed that *p* = *q* = 0.5). The symbol $$\left( {\begin{array}{*{20}{c}} m \\ n \end{array}} \right)$$ means the number of combinations of *m* elements for a set of *n* elements.

Spearman’s rank correlation coefficient was calculated to test an association between the invasion intensity and the observed prevalence of infection (relative to the theoretical expectation). Additional tests were performed to find out whether the dependence between the parameters is possible to approximate with a linear regression equation and whether the regression coefficient is other than zero.

To check the assumption that genotypes are irregularly distributed through the littoral, the χ^2^ test was performed to compare the genotype frequencies between areas of the same littoral level and the average genotype frequencies between different littoral levels.

## RESULTS

The yellow-purple background shell color was the most common in the *L. obtusata* population under study. Its frequency was 55.0% as averaged over the total study period. Periwinkles with a purple background shell color were the second most common (42.4%). Purely orange or yellow shells were relatively rare (2.0 and 0.3%, respectively). Other background shell color variants were detected in trace amounts; their total frequency was 0.3% as averaged over the total study period. Periwinkles with a white-spotted pattern on the shell accounted for 54.2% of the population. Periwinkles with broad longitudinal stripes on the shell occurred in minor amounts; the average frequencies of periwinkles with white, orange, or purple stripes were 1.5, 0.8, and 0.2%, respectively.

Comparisons of the genotype frequencies between individual areas of the same littoral level and the average genotype frequencies between different littoral levels did not detect any significant difference.

Because the phenotypes differed in frequency, their proportions were not the same in groups of periwinkles with particular genotypes. A group with active genes responsible for a background color involving the yellow pigment included predominantly yellow-purple periwinkles (99.3% as averaged over the total study period). A group with active genes responsible for a background color involving the purple pigment included approximately equal portions of periwinkles with a purely purple (43.4%) or yellow-purple (56.3%) background shell color. A group with active genes responsible for a background color involving the orange pigment included periwinkles with a purely orange background shell color almost exclusively (99.9% on average). It should be noted that periwinkles infected with microphallids were detected every year in the groups with active genes for purple and yellow background shell colors, while infected periwinkles were found only in 2003, 2011, 2013, and 2015 in the group with active genes for the orange background shell color because the group was rather small.

**Fig. 1.**
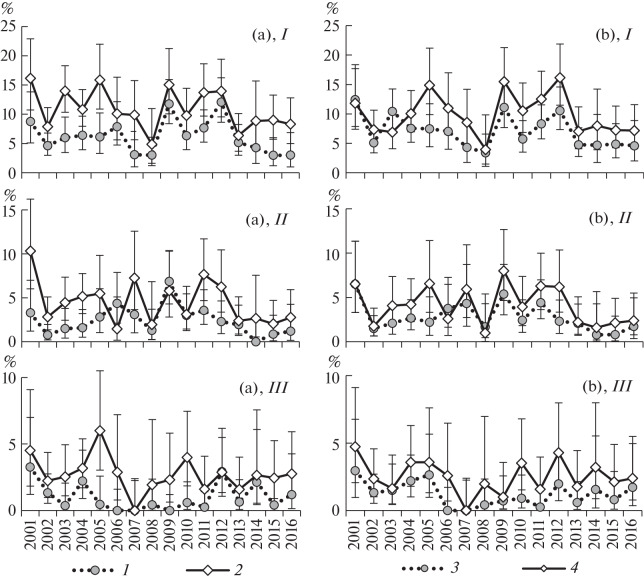
Prevalence of trematode invasion in *L. obtusata* periwinkles with different shell color genotypes. Prevalence was analyzed in (a) periwinkles whose background shell color formed (*1*) with or (*2*) without the yellow pigment and (b) periwinkles that (*3*) had or (*4*) had not a white-spotted pattern on the shell. Trematode invasion was studied for (*I*) the total pygmaeus group and for (*II*) *Microphallus piriformes* and (*III*) *M. pygmaeus* separately. Abscissa, year of observation; ordinate, prevalence of invasion, %. The exact 95% confidence interval is shown for each prevalence value.

**Fig. 2.**
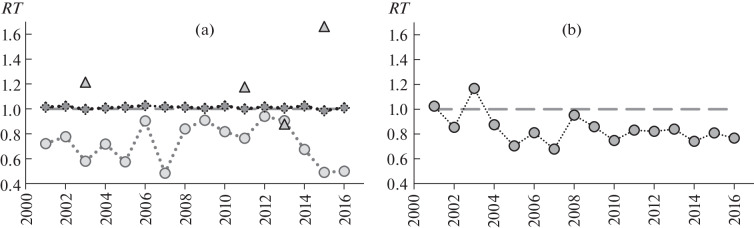
Observed-to-expected prevalence ratio (*RT*) of microphallid infection in *L. obtusata* periwinkles with active genes responsible for (a) background shell coloration and (b) a white-spotted pattern on the shell. Active genes determined the background color with the involvement of the (*1*) yellow, (*2*) purple, or (*3*) orange pigment. Abscissa, year of sample collection.

The prevalence of microphallid invasion in *L. obtusata* with background shell colors based on the yellow pigment was lower than in the group where the yellow pigment was not involved in producing the background shell color in all years of the study (Fig. 1a, *I*). The observed prevalence of invasion varied from 49 to 94% of the expected prevalence, averaging 72.5% (Fig. 2a). A check with the χ^2^ test showed that differences were significant in 9 out of the 16 cases (α < 0.05, Table 1). The probability for a shift to be the same throughout 16 years of observation is vanishingly low, 1.5 × 10^–5^.

No correlation was observed between the prevalence of invasion and the difference between the observed and expected prevalence estimates. Spearman’s rank correlation coefficient *R*_s_ was 0.185 (α $\gg$ 0.05). The regression coefficient obtained for simple linear regression did not differ from zero (α = 0.325).

The prevalence of invasion was analyzed for individual parasite species. The observed prevalence was lower than expected only in the cases of *M. piriformes* and *M. pygmaeus* (67.5 and 48.9% of the expected prevalence, respectively, on average) (Figs. 1a, *II*, *III*; 3a). The observed prevalence of *M. piriformes* invasion was lower than expected in 9 out of the 10 years examined. The difference was significant by the χ^2^ test (α < 0.05) in seven years of the observation period (Table 1), but the probability for the observed or greater differences to occur is low (α = 0.002). In the case of *M. pygmaeus* invasion, differences of the same sign were observed in 14 out of the 15 years (the probability for the observed or greater differences to occur is low, α = 0.001). A check with the χ^2^ test showed that the observed differences were significant in 7 out of the 15 years (α < 0.05, Table 1). No significant difference was observed in the cases of *M. pseudopygmaeus* and *M. triangulatus*; observed-to-expected prevalence ratios were higher or lower than unity at approximately the same frequencies.

The observed prevalence of microphallid infection did not significantly differ from its theoretical expectation in periwinkles with active genes responsible for shell colors involving the purple or orange pigment (Fig. 2a). Observed-to-expected prevalence ratios were nearly unity in periwinkles with active genes for a purple background shell color and were higher or lower than unity at approximately the same frequencies in periwinkles with active genes for an orange background shell color. In all cases, χ^2^ values were lower than the threshold. Thus, activity of the genes responsible for the formation of background shell colors involving the purple and orange pigments did not affect the prevalence of infection.

The prevalence of microphallid invasion in periwinkles with a white-spotted pattern on the shell was lower than in periwinkles without such a pattern in 14 out of the 16 years (Fig. 1b, *I*). The observed prevalence of invasion ranged from 68 to 117% of the expectation, averaging 84.2% (Fig. 2b). Higher-than-threshold χ^2^ values (α < 0.05) were obtained in only 2 out of the 16 years (Table 1), but the probability for similar or greater differences to occur was low (α = 0.002).

Like in the case of background shell coloration, an association was not observed between the prevalence of invasion and the difference between the observed and expected prevalence values. Spearman’s rank correlation coefficient *R*_s_ was 0.129 (α $\gg$ 0.05). A regression coefficient obtained for a simple linear regression equation did not differ from zero (α = 0.392).

An analysis of the prevalence of invasion with particular microphallid species showed that *M. piriformes* and *M. pygmaeus* were responsible for the observed differences, like in the case of background coloration (Figs. 1b, *II, III*; 3b). The observed prevalence was, on average, 82.7 and 63.8% of the expectation for the respective trematode species. In the case of *M. piriformes* infection, the observed prevalence was lower than the expected one in 13 out of the 16 years. A check with the χ^2^ test showed that the difference was significant (α < 0.05) only in 2 out of the 16 years (Table 1). However, the probability for the same or greater differences to occur was low (α = 0.011). In the case of *M. pygmaeus* infection, the observed prevalence was lower than expected in all years. Higher-than-threshold χ^2^ values (α < 0.05) were obtained in only 2 out of the 15 years (Table 1). However, the probability for differences similar in sign to occur at random for 15 years is low, 3.1 × 10^–5^. No significant difference was observed in the case of *M. pseudopygmaeus* and *M. triangulatus* infections.

Periwinkles with different (purple, white, or orange) broad longitudinal stripes on the shell did not significantly differ in the prevalence of infection.

## DISCUSSION

In polymorphic mollusk species, differences in shell coloration between individuals often mark the physiological differences in responses to environmental factors and, in particular, susceptibility to invasion [3, 8, 9]. Differences in the susceptibility to helminthic invasion may be associated with differences in coloration of soft tissues [32–34] and the shell [23, 35]. However, an association does not always occur. The shell color has not been associated with the prevalence of invasion in certain cases [35, 36].

Sergievsky [23] has studied the prevalence of infection in unstriped *L. obtusata* periwinkles with purple, yellow, orange, and brown background shell colors. Periwinkles with or without a white-spotted pattern were considered in the first case, and only spotted periwinkles, in the other cases. The prevalence of invasion in purple and orange periwinkles did not significantly differ from the average prevalence of the population. However, a higher prevalence was observed in yellow periwinkles, and a lower prevalence, in blown periwinkles. A comparison of the prevalence between spotted and unspotted periwinkles with a purple background shell color did not detect any significant difference. The prevalence observed in spotted periwinkles could be higher or lower than the population average, depending on the *L. obtusata* population. Likewise, the prevalence of invasion did not significantly differ between striped and unstriped periwinkles.

**Fig. 3.**
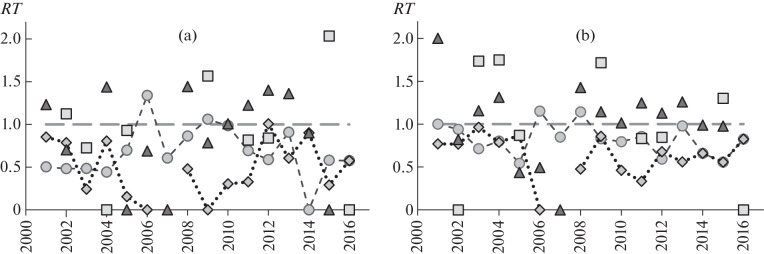
Observed-to-expected prevalence ratio (*RT*) of infection with different microphallid species in *L. obtusata* periwinkles with (a) active genes responsible for a background shell color involving the yellow pigment and (b) a white-spotted pattern on the shell. Microphallids: *1,* *Microphallus piriformes;* *2,* *M. pseudopygmaeus;* *3,* *M. pygmaeus;* *4,* *M. triangulatus*. Abscissa, year of sample collection.

The greatest problem in comparing the results of this work with Sergievsky’s data [23] is related to differences in grouping periwinkles, that is, whether the genotype or the visible shell color (the phenotype) was used for grouping. In view of the differences in grouping, certain reservations should be made when comparing the respective groups. Background coloration is the most problematic to consider in this respect. When grouping is performed by genotype, a particular group includes all periwinkles that display activity of the genes responsible for the inclusion of a particular pigment into the shell. When grouping is performed by phenotype, a similar group includes only periwinkles with a particular visible color (i.e., periwinkles that have active genes responsible for the inclusion of the given pigment in the shell and inactive genes responsible for the inclusion of the other pigments). For example, the purple pigment is included in the shell in purely purple, yellow-purple, white-purple, and certain other periwinkles (see above). Periwinkles with a purely purple background shell color are mostly assigned to the respective group of purple individuals in the case of a phenotype-based grouping.^3^ Based on the frequency of various background shell colors in the population examined in this work, the following genotypic and phenotypic groups are possible to match. Periwinkles with active genes responsible for the inclusion of the purple pigment in the shell (mostly purple and yellow-purple periwinkles) partly correspond to periwinkles that express the “purple” phenotype. Periwinkles with active genes responsible for the inclusion of the orange pigment correspond to a group with the “orange” phenotype. Periwinkles with active genes responsible for the inclusion of the yellow pigment (these were mostly yellow-purple individuals in the habitat examined) correspond to a group with the “brown” phenotype. Fig. 3

In the case of a grouping by the presence or absence of a white-spotted pattern, genotypic and phenotypic groups approximately correspond to each other. The phenotypic groups isolated by Sergievsky included mostly spotted periwinkles with various background shell colors. An association between the presence of a pattern and the prevalence of infection is therefore possible only by comparing the prevalence between unstriped purple periwinkles with a white-spotted pattern and periwinkles lacking a pattern. It should also be noted that the color of broad longitudinal stripes was disregarded in Sergievski’s study. Given that different genes are responsible for stripes of different colors [25, 27], an approximate matching of the genotypic and phenotypic groups is only possible in this case.

With the above reservations, the results of this work generally agree with Sergievsky’s observations [23]. Activities of genes responsible for the background shell colors with the involvement of the purple and orange pigments were not associated with the prevalence of infection. The prevalence was lower than expected in yellow-purple periwinkles in this work, like in the case of periwinkles with the “brown” phenotype. Differences in the prevalence of infection were not observed between striped and unstriped periwinkles in both of the cases. However, the prevalence of infection in periwinkles with a white-spotted pattern was found to be lower than the expectation in this work. These discrepancies most likely arose because differences observed in spotted periwinkles are lower than in periwinkles with a yellow-purple background color and because fewer samples were examined in Sergievsky’s study [23].

It is of interest to note that activity of genes responsible for the pure yellow background shell color is associated with higher susceptibility to infection according to Sergievsky’s data. At the same time, when two gene groups interact to produce a yellow-purple background shell color, the interaction substantially reduces the susceptibility to infection. The finding indicates that caution should be exercised when extrapolating the data from a genotype-based study to the respective phenotypic groups.

Differences in the prevalence of infection in *L. obtusata* with different shell coloration variants may arise because the parasite or, eriwinkle distribution through the littoral is irregular; periwinkles with different genotypes differ in lifespan, the mortality rate of infected individuals, and susceptibility to infection; or other factors act. Special studies are necessary to perform to identify the causes, but are beyond the scope of this work. Certain conclusions are still possible to make based on the available data. Factors associated with an irregular distribution of periwinkles through the littoral are possible to exclude because signs of distribution irregularity were not detected in the region examined. The effects of certain other factors, such as differences in lifespan or the survival of infected periwinkles, seem possible, but unlikely because no clear evidence for differences of the kind was obtained in this work. Genetically determined differences in susceptibility to infection is a far more plausible assumption. Several facts support the assumption. First, genetic factors are known to make a substantial contribution to the susceptibility to helminths in animals and, in particular, mollusks [33, 37–39]. Second, coloration genes may exert pleiotropic effects and thus determine physiological differences in the responses to various environmental factors [3, 8, 9]. Third, genes that are responsible for the susceptibility to infection are known in mollusks [40–47]. Note additionally that differences in the prevalence of infection were observed only for two microphallid species, *M. piriformes* and *M. pygmaeus*. The finding makes it possible to assume that fine biochemical differences between the parasitic species determine the differences in susceptibility.

It should be noted that the difference between observed and expected prevalence values greatly varied between years. Only tentative conclusions are therefore possible to draw from single observations regarding differences in the prevalence of infection between mollusks differing in shell coloration. Stochastic causes are the most probable cause of the variation, but yearly fluctuations in host susceptibility and parasite virulence are also among likely factors. Effects of abiotic environmental factors cannot be excluded as well when the factors somehow affect the survival of mollusks differing in susceptibility to invasion.

Differences in host susceptibility to trematode invasion redistribute the invasion flow through the host population. This redistribution increases the stability of the parasitic system [48]; i.e., one of the phenotypic (or genotypic) groups may have a higher viability in changing environmental conditions and may ensure the preservation of the parasitic system. On the other hand, a higher resistance to invasion confers certain evolutionary advantages on the respective host group. Given parasitic castration caused by trematodes [20–22], the phenotype (genotype) associated with the group will spread through the population, while more susceptible phenotypes (genotypes) will be eliminated gradually. The results of this work indicate that the yellow-purple background shell color and a white-spotted pattern are the phenotypes (genotypes) that have certain selective advantages. If the assumption is true, than more resistant (yellow-purple and spotted) or selectively neutral (purple and orange) phenotypes (genotypes) will accumulate in the population with time. Pure yellow genotypes are more susceptible to invasion and will be preserved in the population to a minor extent, as a result of segregation in crosses between yellow-purple periwinkles. Likewise, a predominance in the population can be expected for periwinkles that have a white-spotted pattern on the shell. Data from long-term observations generally agree with these assumptions. In fact, yellow-purple (55%) and purple (42%) periwinkles predominate in the population under study, while orange (2%) and yellow (0.3%) periwinkles are rare. Periwinkles with a white-spotted pattern are similarly more prevalent (54%) than periwinkles lacking white spots (46%). Periwinkles with an orange background shell color (which were relatively few) do not fit in with the above assumptions, and other factors (e.g., elimination by predators) are most likely responsible for a deficit of periwinkles expressing this phenotype. A far greater predominance could be expected for spotted periwinkles. However, the presence of a spotted pattern is associated with lower changes in susceptibility to infection, while background color genes apparently play a more important role. A more detailed analysis of whether the above hypothesis agrees with the observations is out of the scope of this work and needs special investigation.

## Footnotes

1 A detailed description of *L. obtusata* shell coloration traits with photos is available on the website of the Zoological Institute (St. Petersburg) at https://www.zin.ru/projects/LitPhen/.

2 Parasite descriptions can be found in [12–14, 17, 30].

3 Because only the visible shell color is considered in this case, the phenotypic group may additionally include periwinkles with another genotype. For example, yellow-purple periwinkles with a high intensity of the yellow pigment may be assigned to the “yellow” phenotypic group.
